# Persistent Endothelial Dysfunction in Coronavirus Disease-2019 Survivors Late After Recovery

**DOI:** 10.3389/fmed.2022.809033

**Published:** 2022-02-14

**Authors:** Yi-Ping Gao, Wei Zhou, Pei-Na Huang, Hong-Yun Liu, Xiao-Jun Bi, Ying Zhu, Jie Sun, Qiao-Ying Tang, Li Li, Jun Zhang, Wei-Hong Zhu, Xue-Qing Cheng, Ya-Ni Liu, You-Bin Deng

**Affiliations:** Department of Medical Ultrasound, Tongji Medical College, Tongji Hospital, Huazhong University of Science and Technology, Wuhan, China

**Keywords:** COVID-19, endothelial function, flow-mediated dilation, inflammation, TNF-α

## Abstract

**Background:**

Coronavirus disease 2019 (COVID-19) can result in an endothelial dysfunction in acute phase. However, information on the late vascular consequences of COVID-19 is limited.

**Methods:**

Brachial artery flow-mediated dilation (FMD) examination were performed, and inflammatory biomarkers were assessed in 86 survivors of COVID-19 for 327 days (IQR 318–337 days) after recovery. Comparisons were made with 28 age-matched and sex-matched healthy controls and 30 risk factor-matched patients.

**Results:**

Brachial artery FMD was significantly lower in the survivors of COVID-19 than in the healthy controls and risk factor-matched controls [median (IQR) 7.7 (5.1–10.7)% for healthy controls, 6.9 (5.5–9.4)% for risk factor-matched controls, and 3.5(2.2–4.6)% for COVID-19, respectively, *p* < 0.001]. The FMD was lower in 25 patients with elevated tumor necrosis factor (TNF)-α [2.7(1.2–3.9)] than in 61 patients without elevated TNF-α [3.8(2.6–5.3), *p* = 0.012]. Furthermore, FMD was inversely correlated with serum concentration of TNF-α (r = −0.237, *p* = 0.007).

**Conclusion:**

Survivors of COVID-19 have a reduced brachial artery FMD, which is inversely correlated with increased serum concentration of TNF-α. Prospective studies on the association of endothelial dysfunction with long-term cardiovascular outcomes, especially the early onset of atherosclerosis, are warranted in survivors of COVID-19.

## Introduction

The coronavirus disease 2019 (COVID-19) pandemic caused by severe acute respiratory syndrome coronavirus 2 (SARS-CoV-2) has become a significant challenge to healthcare systems worldwide (1). Though primarily known as a respiratory disease, manifestations from head to toe have been reported in patients with COVID-19 (2). The SARS-CoV-2 attacks the host through angiotensin converting enzyme 2 (ACE2) receptors, which are present in nearly every human organ, including the lungs, heart, kidney, and intestines. Endothelial cells also express ACE2 receptors, representing a potential target for SARS-CoV-2 infection (3). Indeed, previous investigations have confirmed a direct viral infection of endothelial cells and diffuse a vascular inflammation in multiple organs of patients with COVID-19 (4–6). A high prevalence of thrombotic events, including venous thrombosis, pulmonary embolism, and disseminated intravascular coagulation in patients with severe COVID-19 also indicates a dysregulated coagulation system induced by endothelial damage and inflammation during severe SARS-CoV-2 infection (6–8). The endothelial cells participate in the regulation of local and systemic inflammation by generating or responding to cytokines that characterize the cytokine storm in COVID-19, including interleukin (IL)-1, IL-6, IL-8, and tumor necrosis factor (TNF)-α (9), which finally leads to a pro-thrombotic phenotype in patients with severe COVID-19. With increasing evidence of the crucial role of vascular endothelium in the pathophysiology of COVID-19, it has been proposed that endothelial biomarkers and tests of function should be developed and evaluated for their value in risk stratification in patients with COVID-19 and for early detection of long-term cardiovascular complications in convalescent survivors (10).

Recent studies have reported endothelial dysfunction in patients with COVID-19 and convalescent survivors reflected by reduced brachial artery flow-mediated dilation (FMD) in response to hyperemia, which is a non-invasive method to assess systemic vascular endothelial function (11–15). However, these studies have been limited by their short interval between diagnosis and follow-up ranging from 23 to 120 days. Data on endothelial function in survivors of COVID-19 late after recovery are not available. Therefore, the purpose of this study was to examine brachial artery FMD in response to hyperemia with high-resolution ultrasonography in survivors of COVID-19 late after recovery and to explore the potential mechanism underlying the endothelial dysfunction.

## Methods

### Study Design and Participants

This prospective observational study was conducted in Tongji Hospital of Huazhong University of Science and Technology, a tertiary medical center and a designated institute for treating patients with COVID-19. The COVID-19 was diagnosed on the basis of SARS-CoV-2 nucleic acid detection *via* upper respiratory tract swab by a reverse transcriptase-polymerase chain reaction (rt-PCR). Adult patients with confirmed COVID-19 after hospital discharge were invited to participate in the study. Patients were excluded if they had acute conditions such as infection, organ dysfunction, active autoimmune disease, were required hospitalization for other disease, and/or were unwilling to participate. Finally, 86 survivors were recruited between December 2020 and January 2021. Twenty-eight healthy subjects matched for age and sex were recruited as healthy controls. Thirty subjects matched for age, sex, hypertension, diabetes mellitus, smoking, hypercholesterolemia, and coronary artery disease were also recruited as the risk factor-matched controls. All procedures were performed in concordance with the Declaration of Helsinki and International Conference on Harmonization of Good Clinical Practice. The study was approved by the Tongji Hospital Ethics Committee (TJ-C20200156) and informed consent was obtained from each participant before their enrollment in the study.

Clinical characteristics, laboratory results, and medication for acute phase of illness were recorded from the electronic medical system or patient discharge summary of Tongji Hospital. Classification of COVID-19 clinical type was based on the Diagnosis and Treatment Protocol of Novel Coronavirus issued by the National Health Commission of the People's Republic of China (16).

Patients who had fever and respiratory symptoms and had radiologic assessments that showed signs of pneumonia were classifed as Moderate type, while patients who met any of the following criteria were classified as Severe type: (1) shortness of breath, respiratory rate ≥30 times/min; (2) oxygen saturation ≤ 93% at rest; (3) alveolar oxygen partial pressure/fraction of inspiration O_2_ (PaO_2_/FiO_2_) ≤ 300 mmHg; and (4) pulmonary imaging showed significant progression of lesion of >50% within 24–48 h. Lastly, patients who met any of the following criteria were classified as Critical type: (1) respiratory failure requiring mechanical ventilation; (2) shock; and (3) had organ failure and needed intensive care unit monitoring and treatment.

### Assessment of Brachial Artery FMD

Systemic endothelial function was evaluated by measuring brachial artery response to endothelium-dependent stimulus according to the recommendations of European Society of Cardiology (17). All subjects were told to fast and to abstain from drinking coffee or tea before examination. Brachial ultrasound examinations were performed with a subject supine in a quiet, temperature-controlled (22-−24^0^C) environment using a Vivid E95 ultrasound system (GE Medial System, Horten, Norway), equipped with 4–8 MHz 9 L linear array transducer operated by the same examiner throughout the study. The right brachial artery was scanned longitudinally, 5 cm above the antecubital fossa with great care to obtain the maximal vessel diameter and optimal lumen-to-vessel wall interface. When a satisfactory transducer position was found, the skin and the matching position on the probe were marked for reference for later examinations, while the arm was kept in the same position throughout the study. At the same time, anatomical landmarks, like bifurcation of the artery, were also identified and recorded for reference for later examination and measurements. After a resting baseline scan was recorded, the blood pressure cuff placed around the forearm was inflated to a pressure of 200 mm Hg or exceeding >50 mm Hg above systolic pressure if the systolic pressure of the patient was >150 mm Hg for 5 min. Reactive hyperemia was then induced by sudden cuff deflation. The second recording of brachial artery was performed 30 s before the release of the cuff and was continued for a further 3 min after cuff deflation. All recordings were stored in a machine hard disk. The offline analysis was performed by a single observer blinded to the clinical details, time, and intervention. The brachial artery images were input to the computer-assisted analysis platform and the diameter was measured using an automatic echo tracking algorithm. Mean artery diameter was calculated over pre-selected segments at end-diastole and averaged from three consecutive cardiac cycles. The percent change in diameter caused by reactive hyperemia was calculated by dividing the difference from baseline diameter by the baseline value. The Bland-Altman analyses of intra-observer and inter-observer reproducibility for measurements of FMD were previously reported in our laboratory. The mean (95% confidence interval) of FMD difference for intra-observer reproducibility was −0.3(−2.8–2.1) %. The mean (95% confidence interval) of FMD difference for interobserver reproducibility was −0.4(−3.3–2.4) % (18).

### Serum Inflammatory Biomarkers

Peripheral venous blood samples were drawn at least 30 min before vascular ultrasound examination. Blood samples were processed using standardized commercially available test kits for high-sensitivity C-reactive protein (hsCRP), IL-1β, IL-2R, IL-6, IL-8, IL-10, and TNF-α. The hsCRP was detected by immunoturbidimetry method according to the instruction of SEKISUI CHEMICAL CO., LTD (Tokyo, Japan). The IL-1β, IL-2R, IL-6, IL-8, IL-10, and TNF-α were assessed by chemiluminescence immunoassay (CLIA) performed on a fully automated analyzer (Siemens Immulite 1000, DiaSorin Liaison, Vercelli, Italy, or Roche Diagnostics Cobas e602, Basel, Switzerland) according to the instructions of the manufacturers. The CLIA kits for IL-1β (LKL11), IL-2R (LKIP1), IL-8 (LK8P1), IL-10 (LKXP1), and TNF-α (LKNF1) were purchased from DiaSorin (Vercelli, Italy). An IL-6 kit (#05109442 190) was purchased from Roche Diagnostics (Basel, Switzerland). Cut-off values were set according to the kit instructions and were also clinically validated by Laboratory Department of Tongji Hospital. The cut-off values were 10 mg/L, 5 pg/ml, 710 U/ml, 7 pg/ml, 62 pg/ml, 9.1 pg/ml, 8.1 pg/ml for hsCRP, IL-1β, IL-2R, IL-6, IL-8, IL-10, and TNF-α, respectively. The lower limits of detection (LLOD) of hsCRP, IL-1β, IL-2R, IL-6, IL-8, IL-10, and TNF-α were 0.1 mg/L, 5 pg/ml, 5 U/ml, 1.5 pg/ml, 5 pg/ml, 5 pg/ml, and 4 pg/ml, respectively. Values below LLOD were substituted with LLOD for statistical analysis. Participants with TNF-α >8.1 pg/ml were considered to have an elevated TNF-α.

### Statistical Analysis

Categorical variables were expressed as counts and percentage, and continuous variables as mean ± SD or median [interquartile range (IQR)]. Normality was evaluated using the Shapiro-Wilk test. Comparisons among healthy control, risk-factor matched control, and COVID-19 survivor groups were performed using one-way ANOVA with Bonferroni-corrected *post-hoc* tests for normally distributed parameters including body mass index, body surface area, and systolic blood pressure. Kruskal-Wallis test was used among three groups for non-normally distributed parameters, including age, heart rate, diastolic blood pressure, brachial flow-mediated dilation parameters, and inflammatory biomarkers, with Bonferroni-corrected *post-hoc* tests for pairwise comparisons. Mann-Whitney *U*-test was utilized for comparison between TNF-α normal and elevated groups. Comparison of FMD among three clinical types was performed using Kruskal-Wallis test. Wilcoxon test was utilized for comparisons of the data obtained at acute phase and recovery of the illness. Chi-square test or Fisher's exact test was used to compare categorical variables. Associations between FMD and inflammatory biomarkers were analyzed using Pearson or Spearman correlation. A *p* < 0.05 was considered to indicate statistical significance. All statistical analysis was performed using SPSS version 21.0 software (IBM, Armonk, NY, USA).

## Results

### Patient Characteristics

Patient characteristics, brachial artery FMD, and serum inflammatory biomarkers on the day of follow-up are shown in Table 1. A total of 86 patients were enrolled in this study. The median (IQR) age of the COVID-19 group was 58 (39–70) years and 32 patients (37%) were male. According to the Diagnosis and Treatment Protocol of Novel Coronavirus issued by the National Health Commission of the People's Republic of China (16), 45 of 86 patients (52%) were classified as Moderate type of illness, 27 (31%) as Severe type and 14 (17%) as Critical type. The most common chronic cardiovascular conditions in these survivors were hypertension (37%), hypercholesterolemia (19%), diabetes mellitus (16%), and coronary heart disease (15%). Seventy-eight (91%) patients required hospitalization. Among them, 1 patient (1%) underwent an extra-corporeal membrane oxygenation, 6 (8%) underwent mechanical ventilation, and 10 (13%) underwent non-invasive ventilation with positive airway pressure. Inflammatory biomarkers were available in a proportion of patients during hospitalization (Table 2). The IQR interval between the COVID-19 diagnosis and follow-up was 327 (318–337) days. Exertional shortness of breath and chest discomfort were reported in 25 (29%) and 33 (38%) patients, respectively, on the day of follow-up.

**Table 1:** Clinical characteristics, brachial artery flow-mediated dilation, and inflammatory biomarkers of survivors of COVID-19 327 days after diagnosis.

	**Healthy control**	**Risk Factor-Matched control**	**COVID-19**	***p*-value**
	**(*n* = 28)**	**(*n* = 30)**	**(*n* = 86)**	
**Patient characteristics**				
Age, years	56 (37–65)	62 (39–67)	58 (39–70)	0.392
Male, *n*%	10 (36%)	11 (37%)	32 (37%)	0.990
Body mass index, kg/m^2^	23 ± 3	24 ± 3	24 ±3	0.304
Body surface area, m^2^	1.7 ± 0.2	1.7 ± 0.2	1.7 ± 0.2	0.561
Heart rate, bpm	67 (61–81)	69 (63–73)	73 (65–79)	0.119
Systolic blood pressure, mmHg	125 ± 12	126 ± 16	131 ± 18	0.132
Diastolic blood pressure, mmHg	73 (67–82)	72 (67–79)	77 (70–82)	0.228
Oxygen saturation, %	NA	NA	98 (97–99)	NA
Hypertension, *n*%	0 (0%)	10 (33%)^*^	32 (37%)^*^	0.001
Diabetes mellitus, *n*%	0 (0%)	2 (7%)	14 (16%)^*^	0.032
Coronary heart disease, *n*%	0 (0%)	3 (10%)	13 (15%)	0.076
Hypercholesterolemia, *n*%	0 (0%)	9 (30%)^*^	16 (19%)^*^	0.003
**Brachial flow-mediated dilation**				
Baseline diameter, mm	3.6 (3.2–3.9)	3.5 (3.3–4.0)	3.8 (3.2–4.3)	0.266
Diameter during reactive hyperemia, mm	3.8 (3.5–4.2)	3.8 (3.6–4.3)	3.9 (3.4–4.4)	0.940
Percent change in diameter, %	7.7 (5.1–10.7)	6.9 (5.5–9.4)	3.5 (2.2–4.6)^*^^†^	<0.001
**Inflammatory biomarkers**				
High-sensitivity CRP, mg/L	0.7 (0.3–1.6)	0.7 (0.3–1.8)	1.1 (0.6–2.2)^*^	0.039
Interleukin-1β, pg/mL	5.0 (5.0–5.0)	5.0 (5.0–5.0)	5.0 (5.0–5.5)	0.282
Interleukin-2R, U/mL	366 (294–444)	385 (297–482)	370 (282–473)	0.800
Interleukin-6, pg/mL	1.5 (1.5–3.4)	1.5 (1.5–2.8)	1.5 (1.5–2.4)	0.572
Interleukin-8, pg/mL	9.2 (7.0–11.1)	8.1(6.7–10.7)	8.8 (6.8–13.0)	0.482
Interleukin-10, pg/mL	5.0 (5.0–5.0)	5.0 (5.0–5.0)	5.0 (5.0–5.0)	0.371
TNF-α, pg/mL	6.3 (5.5–7.1)	6.2 (5.8–6.8)	6.3 (5.1–8.5)	0.863
Elevated TNF-α, n%	4 (14%)	2 (7%)	25 (29%)^*^^†^	0.027

*Numbers are given as median (interquartile range) or mean ± standard deviation or as case number with percentage in parentheses*.

*NA, not applicable; CRP, C-reactive protein; TNF, tumor necrosis factor*.

* *p < 0.01, vs. healthy control*.

† *p < 0.01, vs. risk factor-matched control*.

**Table 2:** Comparisons of inflammatory biomarkers obtained at an acute phase and after recovery.

	**Number of cases**	**Acute Phase**	**Recovery**	***p*-value**
High-sensitivity CRP, mg/L	*n* = 48	45.1 (6.6–110.7)	1.4 (0.6–2.3)	<0.001
Interleukin-1β, pg/mL	*n* = 47	5.0 (5.0–8.8)	5.0 (5.0–8.3)	0.666
Interleukin-2R, pg/mL	*n* = 47	714 (481–1,154)	410 (336–508)	<0.001
Interleukin-6, pg/mL	*n* = 47	15.9 (4.8–50.8)	1.5 (1.5–2.6)	<0.001
Interleukin-8, pg/mL	*n* = 47	20.4 (10.9–43.5)	9.9 (7.5–14.4)	<0.001
Interleukin-10, pg/mL	*n* = 47	6.7 (5.0–12.5)	5.0 (5.0–5.0)	<0.001
Tumor necrosis factor -α, pg/mL	*n* = 47	10.8 (7.7–15.4)	7.7 (5.8–9.3)	<0.001

*Numbers are given as median (interquartile range)*.

*NA, not applicable; CRP, C-reactive protein; TNF, tumor necrosis factor*.

### Endothelial Function

**Figure 1:**
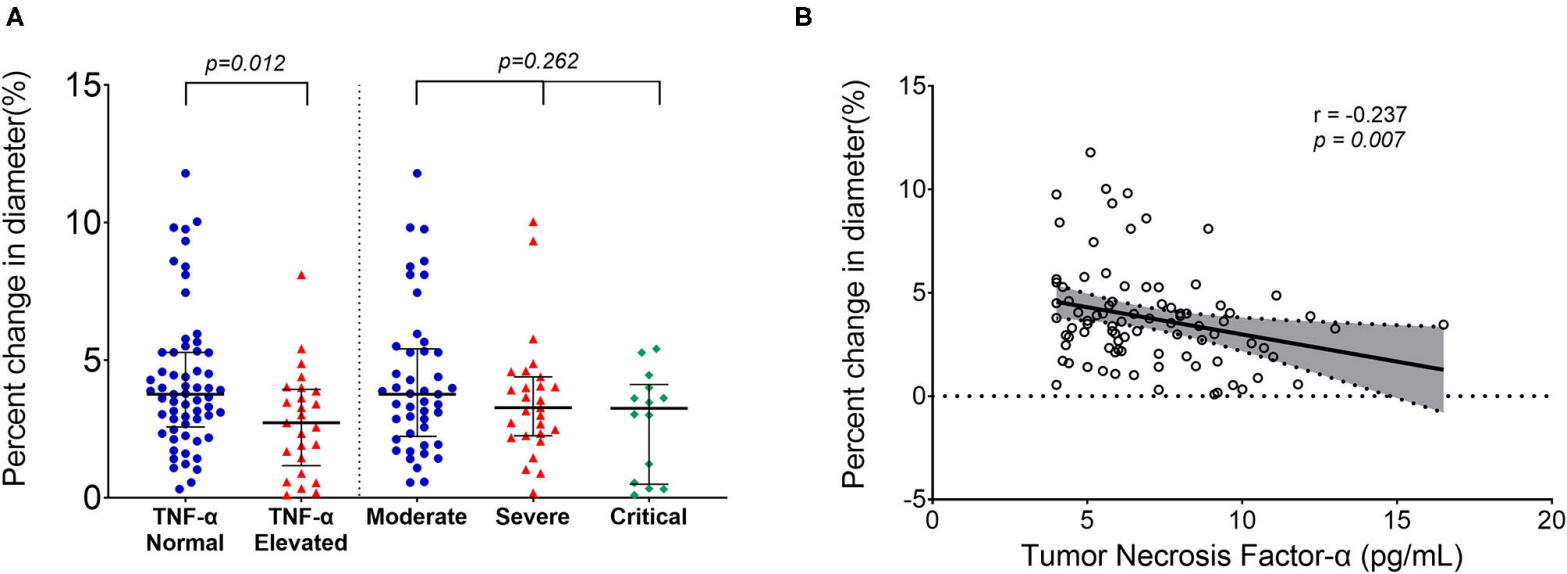
Reduced endothelial function in survivors of COVID-19 late after recovery. No significant differences in brachial artery percent change in diameter existed among groups with different severity of the illness, but it was lower in patients with elevated tumor necrosis factor (TNF)-α than in patients without elevated TNF-α **(A)**. Correlation plots showing the inverse correlation between percent change in diameter and the serum TNF-α level **(B)**. Longer black lines indicate the median and shorter black lines indicate interquartile range in **(A)**. Each dot represents a value. Correlation plots display results from spearman correlation tests and a linear regression line with 95% confidence interval shading in **(B)**.

No significant differences were found in baseline artery diameter among three groups. During reactive hyperemia, the diameter of the brachial artery increased significantly in the control group (*p* < 0.001), survivors of COVID-19 (*p* < 0.001), and risk factor-matched group (*p* < 0.001, Table 1), but FMD was significantly lower in the survivors of COVID-19 than in the healthy controls and the risk factor-matched controls (Table 1). Although no significant differences in FMD existed among groups with different severity of the illness [3.8 (2.2–5.4)% for Moderate, 3.3 (2.3–4.4)% for Severe and 3.2 (0.5–4.1)% for Critical type, respectively, *p* = 0.262, Figure 1A], it was lower in 25 patients with an elevated TNF-α [2.7(1.2–3.9)%] than in 61 patients without an elevated TNF-α [3.8(2.6–5.3)%, *p* = 0.012, Figure 1A]. Furthermore, FMD was inversely correlated with IL-1β, IL-2R, and TNF-α (Table 3 and Figure 1B).

**Table 3:** Correlation analysis between the percent change in brachial artery diameter caused by reactive hyperemia and inflammatory biomarkers in survivors of COVID-19.

	**correlation coefficient**	***p*-value**
High-sensitivity CRP	r = −0.152	0.086
Interleukin-1β	r = −0.186	0.036
Interleukin-2R	r = −0.177	0.046
Interleukin-6	r = −0.167	0.060
Interleukin-8	r = −0.159	0.074
Interleukin-10	r = −0.075	0.399
Tumor necrosis factor -α	r = −0.237	0.007

*NA, not applicable; CRP, C-reactive protein; TNF, tumor necrosis factor*.

### Serum Inflammatory Biomarkers

There were no significant differences among three groups with respect to serum concentrations of IL-1β, IL-2R, IL-6, IL-8, IL-10, and TNF-α. The serum concentration of hsCRP in survivors of COVID-19 was significantly higher than in healthy controls (*p* < 0.01, Table 1). The proportion of participants with elevated TNF-α in survivors of COVID-19 was significantly higher than that of healthy controls (*p* < 0.01, Table 1) and higher than that of risk factor-matched controls (*p* < 0.01, Table 1). Table 2 shows the changes in serum inflammatory biomarkers found between the acute phase and the follow-up 327 days after diagnosis in a proportion of survivors with obtainable biomarker data in acute phase. Of the 47 survivors with cytokine data, 29 (62%) survivors had an undetectable IL-1β and the other 18 (38%) had slightly increased IL-1β in acute phase. In addition, there was no significant difference in median concentrations of IL-1β in acute phase and after recovery (*p* = 0.666, Table 2). Other cytokines and hsCRP, however, decreased significantly (*p* < 0.001, Table 2). The median age (65 years) in this subgroup of 47 survivors was significantly higher than in the overall age of 86 survivors (58 years, *p* = 0.012). However, the clinical type which indicated the severity of the disease at the acute phase of illness showed no significant difference between the two groups (*p* = 0.101). One explanation for the age difference was that seniors were more likely to get tested for cytokine storms in the acute phase of illness since they were more vulnerable to the infection.

## Discussion

Our study showed that survivors of COVID-19 had reduced brachial artery FMD compared with healthy control and risk factor-matched control, which is independent of severity of the illness, but inversely correlated with serum concentration of TNF-α after a median of 327 days from diagnosis. Although concentrations of most inflammatory biomarkers were resolved in survivors of COVID-19, the concentration of hsCRP and percentage of elevated TNF-α in survivors of COVID-19 were still greater than those in healthy and risk factor-matched controls, indicating that a chronic low-grade inflammation could persist late after recovery from COVID-19.

The SARS-CoV-2 attacks the host through the ACE2 receptor, which is also expressed in endothelial cells (3). Previous studies showed that COVID-19 can result in systemic endotheliitis and in widespread endothelial dysfunction *via* direct viral infection of the endothelium or immune-mediated recruitment of immune cells (4, 10). The systemic endotheliitis and endothelial dysfunction in different vascular beds explain the prevalent macro- and micro-vasculopathy in multiple organs in patients with COVID-19. Endothelial dysfunction is characterized by a reduction of the bioavailability of the endothelium-derived vasoactive mediator (mainly nitric oxide) and arterial flow-mediated dilation depends on the ability of the endothelium to release nitric oxide in response to reactive hyperemia or shear stress. Therefore, the brachial artery FMD during reactive hyperemia reflects an endothelial nitric oxide activity and is the marker of endothelial function (19). Unexpectedly, there are only few reports published on FMD measures in patients suffering from COVID-19, so far (11–15, 20, 21). Oliveira et al. reported an endothelial vascular dysfunction assessed by FMD at early stage of illness in patients hospitalized for COVID-19 (20), and Ambrosino et al. documented a significant improvement in FMD in patients with convalescent COVID-19 after a stay of 23 days in hospital (11). A recent study showed reduced brachial artery FMD in 11 young patients with COVID-19 after 3–4 weeks from symptom onset (12), while another study presented lower FMD in survivors of COVID-19 when compared with a control group 4 months after diagnosis (15). In the present study, we demonstrated that brachial artery FMD was reduced in survivors of COVID-19 even 327 days after diagnosis. Furthermore, we also found elevated concentrations of hsCRP, increased percentage of elevated TNF-α, and an inverse correlation between reduced FMD and inflammatory biomarkers in survivors of COVID-19. Although the correlation between FMD and cytokines is not strong, our findings bring new insights into the underlying mechanism of impaired endothelial function in survivors of COVID-19 late after recovery. Previous studies have reported no significant change of IL-1β concentration during the progression of illness in nearly all the patients with either severe or moderate COVID-19 (22). Our study also found that there was no up or downregulation of IL-1β in survivors late after recovery, which further demonstrated the disengagement of IL-1β in the pathogenesis of the disease. On the contrary, TNF-α seems to have participated in the whole course of COVID-19 illness. In acute phase of COVID-19, TNF-α is widely present in blood and infected tissues and acts as an amplifier of inflammation (23). Inflammation is an important factor of endothelial dysfunction, and it has been demonstrated that TNF-α impairs the endothelium-dependent relaxation by affecting nitric oxide production and inducing reactive oxygen species (24–26). It is speculated that during acute SARS-CoV-2 infection, the overwhelming inflammation mediated by TNF-α leads to the systematic injury of endothelial cells, and subsequent endothelial dysfunction remains even after the inflammation subsides (27, 28). The chronic low-grade inflammation indicated by the increased percentage of elevated TNF-α and the elevated concentration of hsCRP in survivors of COVID-19 might partially explain the reduced FMD even nearly 1year after diagnosis. Our findings are consistent with data from previous long-term follow-up studies of Kawasaki disease. Kawasaki disease is characterized by an acute systemic vasculitis that affects infants and young children. Japanese researchers found that the adult patients with history of Kawasaki disease had reduced brachial artery FMD even 24 years after the acute phase of the disease (29). The long-term clinical implication of endothelial dysfunction found in our study is unclear. However, it is well-known that functional arterial changes precede structural abnormalities in atherosclerosis, and endothelial dysfunction is an early event in the pathogenesis of atherosclerosis (30, 31). Thus, the endothelial dysfunction observed in patients with COVID-19 from our cohort and previous studies raises a concern for an increased risk for atherosclerosis in convalescents. Except for the traditional established risk factors for atherosclerosis, such as age, smoking, hyperlipidemia, and hypertension, viral infection has been supposed to be a potential implication in atherosclerosis (32). Endothelial dysfunction emerges as a common mechanism underlying the increased risk of atherosclerosis in people infected with human immunodeficiency virus, hepatitis C virus, and influenza A virus (33–35). Based on the above findings, endothelial dysfunction caused by SARS-CoV-2 becomes a reasonable risk factor in the development of atherosclerosis in survivors of COVID-19. Therefore, prospective longitudinal studies on the association of endothelial dysfunction with long-term cardiovascular outcomes, especially the early onset of atherosclerosis, are warranted in survivors of COVID-19.

There are some limitations in this study. First, no data on FMD values before infection and values at the acute stage of illness were available, which makes the longitudinal comparison of FMD impossible. Second, our study comprised a small sample size of survivors of COVID-19, which limited the generalization of the study conclusion. Third, endothelial function is influenced by multiple factors and, sometimes, it is difficult to distinguish the physiological and pathological arteriosclerosis before any atherosclerotic plaque forms. In our study, COVID-19 was indicated as a potential risk factor rather than an identified pathogenic factor of atherosclerosis. Fourth, hsCRP and cytokines tested in our study could not fully delineate the systemic inflammation. Therefore, a comprehensive long-term follow-up study with larger population is necessary to confirm the impairment of endothelial function and to elucidate its association with SARS-CoV-2 infection.

## Conclusion

This study shows that survivors of COVID-19 have reduced brachial artery FMD that is inversely correlated with concentration of TNF-α after 327 days from diagnosis. Considering the fact that endothelial dysfunction is an early event in the pathogenesis of atherosclerosis, prospective studies on the association of endothelial dysfunction with long-term cardiovascular outcomes, especially the early onset of atherosclerosis, are warranted in survivors of COVID-19.

## Data Availability Statement

The raw data supporting the conclusions of this article will be made available by the authors, without undue reservation.

## Ethics Statement

The studies involving human participants were reviewed and approved by Tongji Hospital Ethics Committee. The patients/participants provided their written informed consent to participate in this study.

## Author Contributions

Y-BD, Y-NL, X-JB, H-YL, and YZ conceived and designed the study. Y-PG, WZ, P-NH, X-QC, W-HZ, Y-NL, and Y-BD collected clinical and ultrasonographical data. Y-PG, WZ, LL, Q-YT, JZ, and JS performed statistical analysis and interpretation of data for the work. Y-PG, WZ, and Y-BD wrote the manuscript. All authors approved the manuscript.

## Conflict of Interest

The authors declare that the research was conducted in the absence of any commercial or financial relationships that could be construed as a potential conflict of interest.

## Publisher's Note

All claims expressed in this article are solely those of the authors and do not necessarily represent those of their affiliated organizations, or those of the publisher, the editors and the reviewers. Any product that may be evaluated in this article, or claim that may be made by its manufacturer, is not guaranteed or endorsed by the publisher.
